# Reflections from COP28: Resisting healthwashing in climate change negotiations

**DOI:** 10.1371/journal.pgph.0003076

**Published:** 2024-03-28

**Authors:** Amiteshwar Singh, Tarek Ezzine, Renzo R. Guinto, Sophie Gepp, Robbie M. Parks, Meelan Thondoo, Poorvaprabha Patil, Kim R. van Daalen

**Affiliations:** 1 Norwich Medical School, University of East Anglia, Norwich, United Kingdom; 2 Health for a Green New Deal, London, United Kingdom; 3 Youth Climate and Health Network, Richmond, California, United States of America; 4 Faculty of Medicine of Tunis, University Tunis El Manar, Tunis, Tunisia; 5 SingHealth Duke-NUS Global Health Institute, Duke-NUS Medical School, National University of Singapore, Singapore; 6 Planetary and Global Health Program, St. Luke’s Medical Center College of Medicine-William H. Quasha Memorial, Quezon City, Philippines; 7 Centre for Planetary Health Policy, Berlin, Germany; 8 Department of Environmental Health Sciences, Mailman School of Public Health, Columbia University, New York, New York, United States of America; 9 MRC Epidemiology Unit, School of Medicine, University of Cambridge, Cambridge, United Kingdom; 10 Barcelona Institute for Global Health (ISGlobal), Centre for Research in Environmental Epidemiology (CREAL), Barcelona, Spain; 11 Kasturba Medical College, Manipal Academy of Higher Education, Manipal, Karnataka, India; 12 Barcelona Supercomputing Center, Barcelona, Spain; 13 Department of Public Health and Primary Care, British Heart Foundation Cardiovascular Epidemiology Unit, University of Cambridge, Cambridge, United Kingdom; 14 Victor Phillip Dahdaleh Heart and Lung Research Institute, University of Cambridge, Cambridge, United Kingdom; PLOS: Public Library of Science, UNITED STATES

At the 28^th^ UN Climate Change Conference (COP28), health emerged as a pivotal focus, with almost 50 Health Ministers and approximately 1,600 health professionals witnessing the inaugural inclusion of a Health Day in official programming. The multidimensional health impacts of climate change—such as changes in the distribution of infectious diseases, changing pollen seasons, and increased heat-related morbidity and mortality—evidently justify the need for this focus [1]. Concurrently, climate action has health co-benefits, including preventing mortality through improved air quality, and improving health through active transport and sustainable, plant-forward diets [2]. The COP28 Declaration on Climate and Health was endorsed by 149 countries, more countries joined the Alliance for Transformative Action on Climate and Health (a WHO-hosted multi-national network dedicated to building climate-resilient, low-carbon healthcare systems), over 200 climate-health events took place, and funding commitments of a total of one billion USD for climate and health was announced. Although separate from the main negotiations, the health day may have catalysed some discussions towards integrating health in COP28 texts. Yet, how health will be integrated in future UNFCCC processes remains unclear.

Whilst the recent acknowledgements of health are welcome progress, it prompts questions about the true motivation behind its initiation and the endorsement from entities profiteering from fossil fuels and the climate crisis, including the COP28 presidency. Healthwashing refers to the misuse of health to advance self-interests (e.g. of companies, governments, organisations) whilst actively contributing to poor health outcomes [3, 4]. Healthwashing activities have previously been highlighted by various commercial determinants of health; private sector activities that impact public health and the enabling of political economic systems and norms [5, 6] Examples of deceitful health claims include food companies advertising unhealth (often ultra-processed and high-sugar) food products as “healthy” by using misleading or unregulated food labels, such as “low-fat”, “source of fibre”, “fewer calories”, “free from artificial flavours” or “natural” [7]. Social media posts with visual and textual cues linked to health (e.g., fruit images, green colours) may likewise be used to instigate a “health halo” that results in consumers overestimating foods’ healthiness [8]. In a similar fashion, the tobacco industry has been aiming to rebrand itself as “part of the solution” by promoting harmful E-cigarettes as “healthy” cigarette replacement products, thereby gaining influence over tobacco and health policy [4, 9]. Corporate political activity has misled decision-makers about products and policies for decades [5, 10].

In the context of climate change, healthwashing refers to anything that increases “the acceptability of initiatives or organisations that minimally advance climate action, whilst ultimately undermining rather than protecting health” [1]. Operating within contemporary neo-colonial, capitalistic structures, healthwashing diverts attention from polluting practises and root causes of environmental health issues, delays regulatory action, diverts action from practical solutions, and undermines public trust. Healthwashing can manifest in the forms of using health messaging to mask potentially harmful activities as being “pro-health”; incorporating health language in climate change negotiations whilst contradicted by previous and current health-harming texts; and making superficial “pro-health” commitments to trade off for “anti-health” decisions elsewhere. Such dynamics are suggested in COP28 negotiations, with countries embracing the Climate and Health declaration, whilst contrarily persisting to allow polluting practises in other key decisions.

Healthwashing’s deceptive nature raises questions about the sincerity of “pro-health” activities at COP28, leaving one to discern between genuine efforts and well-hidden healthwashing. Ultimately, compromises around an unabated fossil fuel phase-out (i.e., only committing to a vague “transitioning away”), and stark shortfalls in financing commitments towards adaptation and loss and damage, risks the health of populations. Health-harming carbon-intensive governments ignore the imperative to limit warming below 1.5°C [11, 12] and continue to expand oil and gas infrastructure by subsidising the fossil fuel industry often due to industry lobbying and economic greed [1]. Neglecting credible climate action will exacerbate health injustice by disproportionately impacting low-income communities, migrants and displaced people, racialised and ethnic minoritised people, as well as sexual and gender minoritised people [13]. Illustratively, it has been estimated that 98% of deaths caused by climate change in 2010 were among populations in the Global South [13].

Healthwashing negatively impacts health globally, regardless of a country’s economic status or progress on achieving climate targets. However, its effects differ substantially between rich and poor countries, influenced by factors like information access, regulations, health disparities, and local factors. Countries with weaker regulatory frameworks around marketing claims, limited markets for health products, and greater health disparities (e.g., in healthcare access, health outcomes, and health determinants such as nutrition and sanitation) [14] are more susceptible to misleading health claims. For example, in settings with limited information access, communities may struggle to discern genuinely healthy products and policies from deceptive ones [15]. Left unchecked, healthwashing will exacerbate existing health gaps and environmental injustices.

Recognising these challenges, the health community should respond strategically by collaboratively and actively integrating its influence across climate change negotiations, breaking free from siloed approaches. Regular expert reviews of health-related climate commitments are crucial for holding governments and non-state actors accountable and distinguishing their efforts from healthwashing practices. Commitments should be specific, measurable, and time-bound, addressing upstream determinants like fossil fuel burning, and integrated into national climate plans—including explicit means of implementation and support such as just financing mechanisms. Benchmarks can be used to compare and quantify performance against standards or best practices. Transparency in reporting and access to data from governments and non-state actors is hereby essential to foster accountability, increase public trust and allow independent review of the alignment between commitment and actual practice. Importantly, to identify and prevent healthwashing, evaluation of progress should extend to the contribution of relevant actors to industries harmful to health or the planet, alongside broader fulfilment of internationally agreed climate targets (i.e., assessment of hidden trade-offs). Fundamentally, actors threatening the integrity of the health space, such as the fossil fuel industry, must be prohibited from entering, misusing and influencing its discourse [3].

The health community also needs to enhance its capacity to recognise and confront healthwashing, which includes addressing loopholes in negotiations (e.g., transitional fuels). Aligned with the emerging study of the commercial determinants of health [5], health scholars and practitioners should further unveil, identify, and analyse climate-related healthwashing and its implications in UNFCCC processes and beyond. This endeavour should include clear definitions, up-to-date metrics, and identifiers of climate-related healthwashing, and mechanisms for information dissemination to prohibit further healthwashing.

Perpetrators of—and those benefiting from—the climate crisis are co-opting health to advance self-interests whilst endangering planetary health. Having had made progress over the years, it is the health community’s duty to resist the hijacking of health justice. The state of global public health is a political and moral choice. The global health community must be proactive in identifying and resisting acts of healthwashing and thereby stand as a pillar for climate and health justice.
